# 

**Published:** 1827-01

**Authors:** 


### Contents

OF

### No. 11. Vol. V. (NEW SERIES) for JANUARY, 1827.

### [*No.* 27, *ANALYTICAL SERIES.*]

Art. I.

A Treatise on Diet, disordered state of the Digestive Organs, &c. By John A. Paris, M.D. &c. &c. .......... 1

II.

Elementary Treatise on Diagnosis, Prognosis, and Therapeutical Indications; a Course of Clinical Medicine. By M. L. Rostan, M.D..... 51

III.

Transactions of the Medico-Chirurgical Society of Edinburgh, Vol. II. continued from No. X. p. 408.

1. Dr. Fairbairn on Purpura Hæmorrhagica.......... 61
2. Observations on the Physiological principle of Sympathy, with reference to the peculiar Doctrines of Mr. Charles Bell. By W. P. Alison, M.D. with Critical Observations .......... 63
3. Dr. James Pitcairn on Empyema successfully treated by Paracentesis Thoracis .......... 78
4. Dr. John Gairdner on Carditis, with unusual Symptoms .......... 79
5. Mr. Charles Miller on Hydrocephalus Chronicus, with unusual Symptoms, and the Appearances on Dissection.......... 81
6. Notes of some Remarkable Cases. By Dr. James Molleson. Stricture of the Ileum and Artificial Anus,.......... 83
7. On the High Operation of Lithotomy. By Dr. George Ballingall, .... 83
8. On Disorganization of the Stomach in Infants. By Dr. J. Gairdner .. 86

IV.

Medical Tour in Italy. By Dr. Louis Valentin. 2d Edition, enlarged.. 88

V.

Mr. Benjamin Travers on Constitutional Irritation. (3rd and last Article) 101

VI.

Principles of Dental Surgery; exhibiting a New Method of Treating Diseases of the Teeth and Gums, &c. By Leonard Kœcker, M.D. Surgeon-Dentist, &c.......... 129

*Concluded at page* 213.

VII.

### Quarterly Periscope

OF

### PRACTICAL MEDICINE;

BEING

*THE SPIRIT OF THE MEDICAL JOURNALS,*

FOREIGN AND DOMESTIC.

PART I.

### PERISCOPE OF HOSPITAL REPORTS,

*ENGLISH AND FOREIGN,*

WITH COMMENTARIES.

1. A Memoir on the Anatomical Characters of Chronic Gastritis. By M. Andral, M.D.—*(La Charité)*

   Part I. Alterations of the Mucous Membrane.......... 145

Part 2. Alterations of the other Tissues of the Stomach.......... 153
Cases in Illustration .................................... 159
2. Dr. Chambers on the Nature and Treatment of Fever, with Cases.—*(St. George's Hospital)*.............................. 161
3. Ligature of the Subclavian Artery. By M. Dupuytren—*(Hotel Dieu)* 163
4. Wound of the Carotid Artery. By Benjamin Travers, Esq.—*St. Thomas's Hospital)*.................................. 165
Mr. Fleming's Case of Wound and Ligature of the Carotid Artery, before Mr. Abernethy's Operation.. ........................ 167
5. M. Paillard on Injuries of the Head, with Cases—*(St. Louis Hosp.)*.. 169
Remarks on Injuries of the Head ............................ 171
6. Mr. Mackenzie on the Different Kinds of Opththalmia—*(Eye Infirmary, Glasgow)*.................................... 173
7. Dr. Dance's Memoir on Morbid Invaginations of the Intestines, with Cases—*(Hotel Dieu)* ................................ 176
Historical Review of this Disease, with Cases and Criticisms ...... 180
8. Dr. Mc Andrew on Chorea—*(S. London Dispensary)* ............. 189
9. M. Lisfranc's Clinical Reports—*(La Pitié)* .................... 189
1. Extirpation of the Eye.................................. 190
2. Hernia and Perforation of the Stomach ..................... 190
3. Treatment of Herpes Zoster ................................ 191
4. On Wounds of the Scalp, with Critical Remarks ............. 191
10. Dr. Gregory on the most Effectual Method of Vaccinating—*(Small-Pox Hospital)*....................................... 192
11. Mr. Dalziell on Aneurism at the Bend of the Arm, from Venesection 193
Cases in St. George's and the Westminster Hospitals, with Criticisms thereon.............................................. 194
12. M. Paillard on the Natural Cure of Wounds of the Intestines, with Cases 196
Remarks on this Subject ................................... 197
13. Dr. Hewett on the Treatment of Fever with Follicular Ulceration—*(St. George's Hospital)*................................. 198
14. Mr. Earle on Local Irritation, occasioning great Constitutional Disturbance—*(Bartholomew's Hospital)*.......................... 199
15. Drs. Hawkins and Chambers on Purpura Hæmorrhagica—*(St. George's and Middlesex Hospitals)* ............................... 200
16. Memoir on Abscesses in the Liver, with Numerous Cases. By M. Louis —*(La Charité)* ...................................... 201
17. Mr. Stone on Polypus Uteri—*(Brownlow-street Lying-in Hospital)* .. 210
18. Messrs. Monot and Jobert on Tetanus, with Cases—*(Hospitals St. Louis and St. Antoine)* ................................. 211
19. Postscript to Dental Surgery................................ 213

### VIII.

### PART II. ANALECTA.

1. Remarkable Disease of the Heart, Lungs, and Liver .............. 215
2. Can the Blood be the Seat of Diseases? Experiments on this Question by M. Segalas.......................................... 218
3. Regeneration of a Sphacelated Urethra ........................ 220
4. Rare Cases in Surgery. By Professor Scarpa—
1. Enormous Collection of Milk in the Mamma .............. 221
2. A Foreign Body in the Rectum ........................... 222
5. Dr. Hamilton on a Modification of Sore Throat in Children ........ 223
6. Propensity to Homicide, Infanticide, and Suicide .................. 226
7. Hyosciamus and Camphor in Gonorrhœa ....................... 227
8. Case of Cherry-stone lodged in the Lungs ....................... 228
9. Dr. Brown on a New Mode of Treating Ague...................... 229
10. Curious Case of Encephalic Phlebitis ........................... 230

11. Mr. Chevalier on Belladonna........ 231
12. On Compression in Poisoned Wounds........ 232
13. Abdominal Dropsy with Pregnancy........ 234
14. Great Disease of Brain, without the usual Symptoms........ 234
15. Case of Excision of the Head of the Humerus........ 235
16. Case of Want of Pericardium........ 235
17. Remarkable Cases of Dysphagia........ 236
18. Dr. Bardsley on Diabetes Mellitus........ 238
19. Clinical Cases. By Dr. Bland—
1. Cerebral Hæmorrhage........ 238
2. Case of Pharyngitis........ 239
20. Extravasation of Blood on the Spinal Marrow........ 239
21. Dr. Kruger and Mr. Sym on Perforations of the Stomach........ 240
22. Dr. Ballardini on Epilepsy........ 241
23. On the Propriety of Employing Ergot of Rye........ 242
24. Mr. Donaldson on Poisoning by Hyosciamus........ 242
25. Sulphate of Quinine in Friction........ 243
26. Observations on Bronchocele........ 243
27. Broussais and Clutterbuck in Danger........ 244
28. New Remedy for Incontinence of Urine........ 244
29. Instance of Wound of the Heart not quickly Mortal........ 245
30. Curious Cases of Sympathetic Epilepsy........ 245
31. Fistula in Perineo cured by an Operation........ 245
32. Lunar Caustic in Inflammation of Absorbents........ 246
33. Severe Punctured Wound of Posterior Tibial Artery........ 247
34. Mr. Mayo's Experiments on the Bile........ 247
35. Remarkable instance of Vicarious Menstruation........ 248
36. Mr. Fielding on Silk-worm-gut Ligatures........ 249
37. Mr. Boyle on Moxa in Phlegmatia Dolens........ 250
38. Case of Sudden and Mysterious Death........ 251
39. Remarkably severe Gun-shot Wound, with Remarks........ 251
40. Dr. Duparque on Subcarbonate of Iron........ 252
41. Prince Hohenloe in Panton Square........ 253
42. Remarkable Case of Chronic Dyspnœa........ 254
43. Empyema, with uncommon Phenomena........ 255
44. Spectral Illusions; or the Influence of the "Digestive Organs" on Mr. Abernethy's Optics........ 256
45. Ergot of Rye in Uterine Hæmorrhage........ 257
46. Curious Case of Vescial Fistula, with Remarks........ 257
47. Mr. Lizars on Amputation of the Lower Jaw........ 258
M. Dupuytren on the same Operation........ 258
48. Orthopœdia........ 258
49. Case of Retroversio Uteri. By Mr. Wise........ 259
50. Remarks on Cases of Phagedenic Ulceration........ 259
51. Remarks on Mr. Annesley's Opinions respecting Mercury........ 260
52. On Chronic Gastritis. By Mr. Brown........ 261
53. Mr. Tyrrel's Case of Compound Dislocation........ 262
54. Slicing Structure in Search of Function........ 262
55. Mr. Abernethy on Spinal Diseases, with Remarks........ 263
56. On the Trephine in Injuries of the Head........ 263
Criticisms on this point of Surgery........ 264
57. On Spasms of Canals, with Remarks........ 265
58. Mr. Lawrence's Utopia........ 266
59. Puzzling Points for a Court of Conscience........ 268
60. Exhumation and Fumigation—New Resurrection Company........ 268
61. College of Surgeons—"Youths of Fifteen"........ 268
62. Sir A. Carlisle's Iron Blisters........ 269
63. Important Anatomical Discovery........ 270
64. New Patents—Vituperation Company........ 270

65. Non-Jurors—Medical Superstition ........ 270
66. Pregnancy complicated with Hydatids ........ 270
67. Cure of Phthisis by Mercury ........ 271
68. New Operation for Piles ........ 271
69. Fractures of the Cervix Femoris ........ 272
70 Internal Strangulation ........ 272
71. Phlogosis of the Retina ........ 272

### IX.

### EXTRA-LIMITES.

I. Mr. Lizars's Observations on Lithotomy; with Critical Observations on certain Writings of Dr. Monro and Mr. Allan ........ 278
II. Medical Jurisprudence—
1. Regulations respecting Medical Degrees at Aberdeen ........ 281
2. Ditto Ditto Ditto St. Andrew's ........ 282
III. Hunterian Museum—Fellows and Licentiates—
1. Dr. Ager's Circular Letter ........ 283
2. Dr. Ager's Statement of Facts ........ 284
3. Dr. Macleod's Letter to Sir Henry Halford ........ 285
4. Remarks on the foregoing Subject, and Answer to the Letter of Socius in the Medical Journal ........ 288
IV. Pathology and Treatment of Croup ........ 289
V. Qualifications of Army Medical Officers ........ 291
VI. Reclamation of Dr. Barry ........ 292
VII. Regulations respecting Medical Degrees of the University of Glasgow 294
VIII. Experiments of M. M. Rigot and Trousseau on the Changes which take place Post Mortem ........ 295
IX. Case of Poisoning by Bitter Almonds ........ 298
X. Dr. Anderson on the use of Tobacco in Tetanus ........ 298
XI. Fracture of the Neck of the Thigh-Bone ........ 299
XII. Fracture of the Patella ........ 299
XIII. Retrospective Review of the Medical Periodical Press of Great Britain. (No. 1.) ........ 300
The Medical Essays and Observations of Edinburgh ........ 301
1. Combination of Ascites with Pregnancy ........ 302
2. Dr. Whytt's Anticipation of some of the Doctrines of Dr. W. Philip ........ 303
3. Remedy for Obstructed Menses ........ 303
4. Mercurial Inunction in Tetanus ........ 304

### X.

Bibliographical Record ........ 305

### XI.

### MEDICAL INTELLIGENCE.

1. American Edition of the Medico-Chirurgical Review, to complete Sets, imported by Mr. Highley ........ 309
2. Dr. Barry's Invitations to the Medical Societies of London, and the Discussions there ........ 310
3. Queries addressed to Mr. Lizars ........ 310
4. Dr. Spurzheim and the Cock of the North ........ 310
5. Various Literary Notices ........ 311
6. List of New Subscribers ........ 312
7. Prize offered for the best Hospital Report ........ 312

